# Calculated plasma volume status predicts outcomes after transcatheter aortic valve implantation

**DOI:** 10.1136/openhrt-2020-001477

**Published:** 2020-12-23

**Authors:** Annette Marie Maznyczka, Mohamad Barakat, Omar Aldalati, Mehdi Eskandari, Ann Wollaston, Vasileios Tzalamouras, Rafal Dworakowski, Ranjit Deshpande, Mark Monaghan, Jonathan Byrne, Olaf Wendler, Philip MacCarthy, Darlington Okonko

**Affiliations:** 1 British Heart Foundation Glasgow Cardiovascular Research Centre, Institute of Cardiovascular and Medical Sciences, University of Glasgow, Glasgow, U.K, Glasgow, UK; 2 Cardiology, King's College Hospital, London, UK; 3 King's College London British Heart Foundation Centre of Excellence, School of Cardiovascular Medicine and Sciences, James Black Centre, London, UK; 4 Cardiothoracic Surgery, Kings College Hospital Kings Health Partners London UK, London, UK

**Keywords:** transcatheter aortic valve replacement, heart failure, heart valve diseases

## Abstract

**Objectives:**

Congestion can worsen outcomes after transcatheter aortic valve implantation (TAVI), but can be difficult to quantify non-invasively. We hypothesised that preprocedural plasma volume status (PVS), estimated using a validated formula that enumerates percentage change from ideal PV, would provide prognostic utility post-TAVI.

**Methods:**

This retrospective cohort study identified patients who underwent TAVI (2007–2017) from a prospectively collected database. Actual ([1-haematocrit] × [a + (b × weight (Kg))] and ideal (c × weight (Kg)) PV were quantified from equations where a, b and c are sex-dependent constants. Calculated PVS was then derived (100% x [(actual – ideal PV)/ideal PV]).

**Results:**

In 564 patients (mean age 82±7 years, 49% male), mean PVS was −2.7±10.2%, with PV expansion (PVS >0%) evident in 39%. Only logistic European System for Cardiac Operative Risk Evaluation (EuroSCORE) independently predicted a PVS >0% (OR 1.85, p=0.002). On Cox analyses, a PVS >0% was associated with greater mortality at 3 (HR 2.29, 95% CI 1.11 to 4.74, p=0.03) and 12 months (HR 2.00, 95% CI 1.23 to 3.26, p=0.006) after TAVI, independently of, and incremental to, the EuroSCORE and New York Heart Association class. A PVS >0% was also independently associated with more days in intensive care (coefficient: 0.41, 95% CI 0.04 to 0.78, p=0.03) and in hospital (coefficient: 1.95, 95% CI 0.48 to 3.41, p=0.009).

**Conclusion:**

Higher PVS values, calculated simply from weight and haematocrit, are associated with greater mortality and longer hospitalisation post-TAVI. PVS could help refine risk stratification and further investigations into the utility of PVS-guided management in TAVI patients is warranted.

**Key questions**

**What is already known about this subject?**

- Congestion predicts adverse outcomes in many clinical settings, especially perioperatively.
- Many patients with cardiac disease remain haemodynamically overloaded despite clinical euvolaemia.
- Plasma volume status (PVS) can be estimated using a validated formula, incorporating simple clinical indices (weight and haematocrit), which enumerates percentage change from ideal plasma volume.

**What does this study add?**

- In transcatheter aortic valve implantation (TAVI) patients, congestion (as signified by PVS >0%) associates with mortality.
- In TAVI patients, PVS >0% associates with longer duration of hospitalisation.

**How might this impact on clinical practice?**

- Calculated PVS may have additive value if incorporated into risk stratification models.
- PVS-guided fluid management in TAVI patients warrants further investigation.

## Introduction

Transcatheter aortic valve implantation (TAVI) is the standard of care for patients with severe aortic stenosis who are at high or prohibitive surgical risk. In patients at low or intermediate risk, TAVI is at least non-inferior to surgical aortic valve replacement.^1 2^ TAVI use is increasing worldwide^3^ and the move towards conscious sedation, smaller delivery systems and improved vascular closure techniques have facilitated earlier discharge with potential cost savings. Despite these refinements, however, in-hospital and 1-year mortality rates after TAVI are ~3% and 22%, respectively,^3^ underscoring the need for novel remediable markers of adversity after TAVI.

Congestion is a powerful predictor of adverse outcomes in many clinical settings, especially perioperatively^4 5^ and heart failure before TAVI is associated with adverse clinical outcomes.^6^ While overt congestion is easy to detect, many cardiac patients are haemodynamically overloaded despite clinical euvolaemia (covert congestion), and the prognosis for these individuals is nearly as bad as for those with overt oedema.^7–9^ Novel markers of congestion that can accurately detect subclinical congestion have potential to provide incremental prognostic information, when considered alongside other predictors of clinical outcomes in TAVI patients.^10–13^ Accurate markers of congestion may also help guide diuretic therapy prior to TAVI.

Plasma volume (PV) expansion underlies systemic congestion in cardiac patients and can be objectively estimated using validated equations based on weight and haematocrit.^14 15^ In prior studies, calculated PV levels were shown to mirror those measured using gold standard radioisotope assays.^16 17^ Moreover, relative PV status (PVS), a measure of the degree to which patients have deviated from their ideal PV, predicted mortality in various heart failure cohorts,^16–19^ and in patients undergoing coronary artery bypass grafting.^20^

In the present analysis, we tested the hypothesis that preprocedural cardiac congestion, detected by a higher preprocedure PVS, would predict a longer duration of hospitalisation, longer stay on the intensive care unit (ICU) and a greater risk of mortality after TAVI.

## Methods

### Population

Consecutive patients who underwent TAVI at King’s College Hospital London between August 2007 and March 2017 were identified from an approved prospectively collected national registry database, which was retrospectively studied. Patient demographics, procedural characteristics, duration of hospitalisation and procedural outcomes were recorded prospectively onto the registry, and reported according to Valve Academic Research Consortium-2 criteria.^21^ All-cause mortality was ascertained from the registry and verified from hospital information systems that are regularly updated with deaths from the Office of National Statistics. The need for individual patient informed consent was waived by the local research office as this was a retrospective analysis using a de-identified database.

Each patient was selected for TAVI by a multidisciplinary Heart Team, attended by interventional and imaging cardiologists, cardiothoracic surgeons and cardiac anaesthetists. The patients selected for TAVI were either formally turned down for surgical aortic valve replacement by two cardiothoracic surgeons, or were deemed too high-risk for surgical aortic valve replacement. From 2007 to 2012, patients were routinely monitored on ICU post-TAVI. After October 2012, patients were cared for on a level-two unit post-TAVI and not in ICU. Of the 576 patients in the database, 10 were excluded because of missing weights and two were excluded due to missing haematocrits. Thus, 564 patients were included in this study. In-hospital weights and haematocrits documented just before TAVI were used to calculate PVS.

### PV equations

Actual PV was calculated with the following equation, derived by curve-fitting techniques using the participants' haematocrit and weight compared with PV values measured with the gold standard radiolabelled albumin assay.^16^

actualPV=(1−haematocrit)×(a+[b×weight(Kg)])

where haematocrit is a fraction, and a=1530 in males and 864 in females, and b=41 in males and 47.9 in females.

Ideal PV was calculated from the following established formula^17^ :

idealPV=c×weight(Kg)

where c=39 in males and 40 in females.

Relative PVS, an index of the degree to which patients have deviated from their ideal PV, was subsequently calculated from the following equation:

PVS=([actualPV–idealPV]/idealPV)×100%.

### Statistical analysis

Data are presented as proportions (%), mean±SD or medians (IQR. Intergroup comparisons were made using a Student’s t-test, Mann-Whitney U test, Pearson χ^2^ test or Fisher’s exact test as appropriate. The distribution of continuous variables was visually assessed.

Associations between PVS and baseline covariates, lengths of stay and postprocedural complications were evaluated using univariable and multivariable linear or logistic regression. For baseline correlates of PVS, multivariable regression only included covariates that differed between patients who did and did not have PV expansion (ie, PVS>0%), and that were not directly related to the European System for Cardiac Operative Risk Evaluation (EuroSCORE) so as to minimise collinearity. For the association of PVS to lengths of stay and complications, multivariable models only adjusted for *a priori* determined clinically relevant covariates (EuroSCORE and NYHA (New York Heart Association) class) to minimise collinearity and prevent model overfitting. A p<0.20 was used to enter and retain covariates in multivariable models. The validity of linear and logistic regressions was verified by analysis of model residuals, linearity condition, testing for heteroscedasticity and the absence of interaction and multicollinearity. k-fold cross-validation was used to quantify overfitting. As lengths of stay were not normally distributed, bootstrap linear regression with 1000 bootstrap samples was performed to model these endpoints.

Association between PVS and all-cause death was determined using univariable and multivariable Cox proportional hazards regression. Multivariable Cox models only included PVS, EuroSCORE and NYHA class to minimise collinearity and prevent model overfitting. The significance levels for chi-square (likelihood ratio test) were calculated. The validity of Cox models was verified by assessing the proportionality of hazards, log-linearity, and absence of interaction and multicollinearity. Kaplan-Meier cumulative survival plots were constructed for visualisation and assessed using the log-rank test.

Calculated PVS was assessed as both a continuous and categorical variable. The shape of the association between PVS and outcomes was investigated by constructing restricted cubic spline plots on the PVS function. The PVS cut-off that best discriminated mortality was determined using receiver operating characteristics analysis. In this, sensitivity and specificity were of equal importance, therefore, the optimal PVS cut-off was the one giving the highest Youden index. The incremental predictive ability of PVS was evaluated by calculating the continuous net reclassification improvement and the integrated discrimination index. To facilitate this for the analysis of hospital stay, patients were split by the median days in hospital into long-stayers if they stayed above the median, or short-stayers if they did not.

Only 1.6% of the total data was missing and survival analyses with and without imputation showed consistent results. No adjustments for multiple statistical comparisons were made. Data were analysed using SPSS (V.25.0, SPSS, IBM) and STATA (V.12, StataCorp). A two tailed p<0.05 was considered statistically significant.

## Results

### Population and procedural characteristics

**Figure 1:**
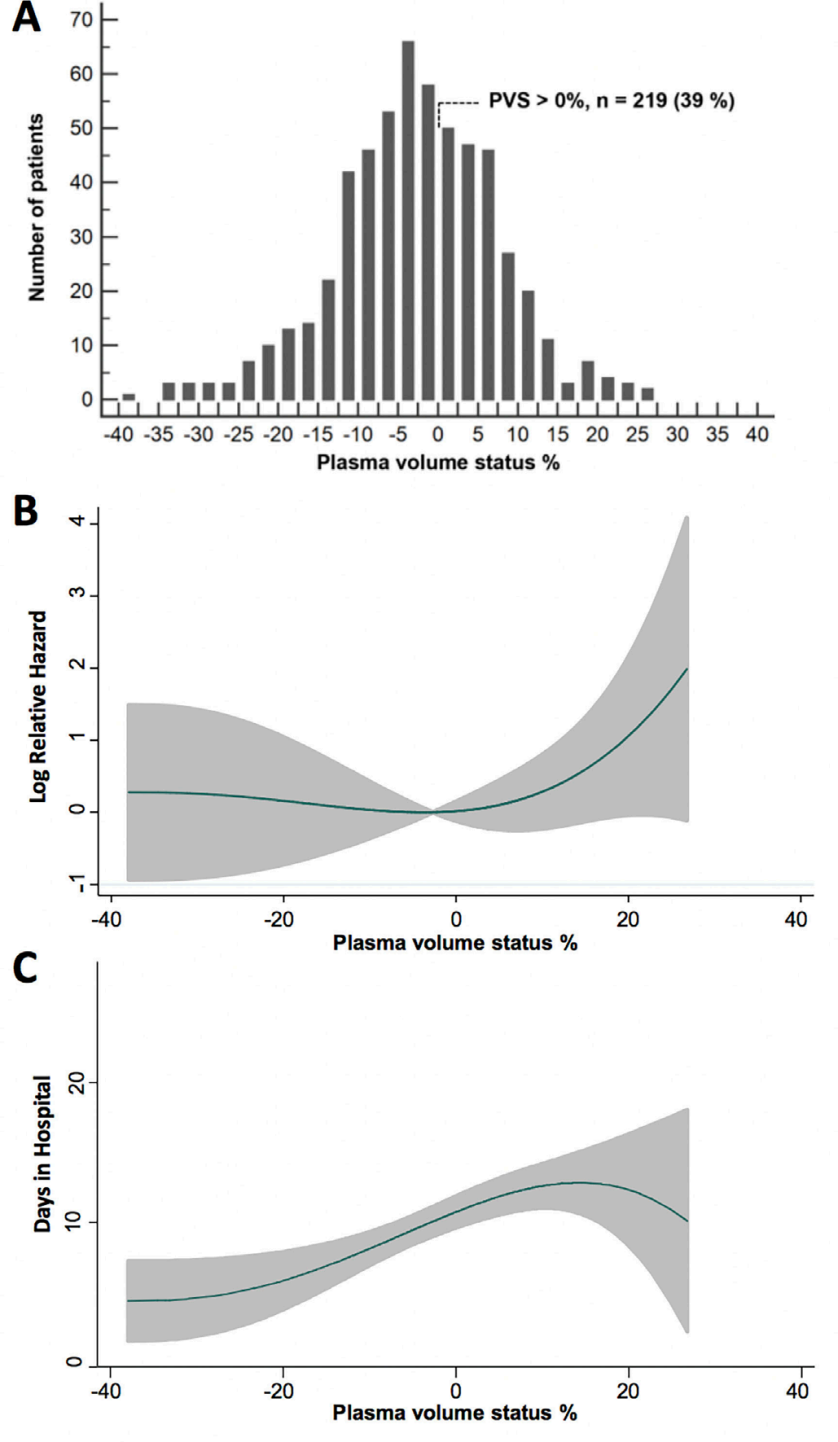
Distribution of plasma volume status (PVS) (A), and shape of relation of PVS to in-hospital mortality (B) and days in hospital (C) grey shaded area in (B) and (C) denotes the 95% CI.

The baseline and procedural characteristics of the 564 TAVI patients are shown (table 1). Calculated PVS appeared to have a normal distribution and ranged from −38.0% to 26.8% (figure 1A).

**Table 1:** Baseline and periprocedural characteristics stratified by PVS

Variable	All patients (n=564)	PVS ≤0% (n=345)	PVS >0% (n=219)	P value
Age, years	82±7	82±7	83±7	0.23
Male sex	277 (49)	160 (46)	117 (53)	0.10
Body mass index, kg/m^2^	26 (23 to 30)	28 (25 to 32)	24 (21 to 27)	**<0.001**
Logistic EuroSCORE	14 (10 to 23)	13 (9 to 21)	16 (10 to 26)	**0.002**
Canadian cardiovascular society angina class ≥III	6 (1)	3 (1)	3 (1)	0.68
NYHA class ≥III*	365 (65)	218 (63)	147 (67)	0.36
Diabetes mellitus	125 (22)	76 (22)	49 (22)	0.92
Hypertension*	440 (79)	274 (79)	166 (77)	0.41
Renal failure†*	17 (3)	6 (2)	11 (5)	**0.03**
Pulmonary disease*	136 (24)	86 (25)	50 (23)	0.55
Peripheral vascular disease	118 (21)	61 (18)	57 (26)	**0.02**
Atrial fibrillation/ flutter	151 (27)	101 (29)	50 (23)	0.09
Prior cerebrovascular accident*	42 (8)	26 (8)	16 (7)	0.94
Prior myocardial infarction*	49 (9)	31 (9)	18 (8)	0.74
Prior cardiac surgery*	126 (22)	71 (21)	55 (25)	0.20
Previous percutaneous coronary intervention*	99 (18)	67 (19)	32 (15)	0.15
Left ventricular ejection fraction, n (%)*				0.29
Good (ejection fraction ≥50%)	387 (69)	241 (71)	146 (68)	
Fair (ejection fraction 30%–49%)	149 (27)	85 (25)	64 (30)	
Poor (ejection fraction <30%)	22 (4)	16 (5)	6 (3)	
Coronary artery disease	171 (30)	98 (29)	73 (33)	0.24
Triple vessel disease	46 (8)	30 (9)	16 (7)	0.54
Aortic valve pathology*				0.53
Aortic stenosis	533 (95)	328 (96)	205 (95)	
Aortic regurgitation	13 (2)	6 (2)	7 (3)	
Mixed aortic valve disease	14 (3)	9 (3)	5 (2)	
Severe aortic calcification*	287 (55)	190 (59)	97 (48)	**0.02**
Mean aortic gradient, mm Hg*	42±16	41±16	44±16	**0.02**
Peak aortic gradient, mm Hg*	75±25	73±25	79±25	**0.01**
Aortic valve area, cm^2^*	0.7 (0.6 to 0.8)	0.7 (0.6 to 0.9)	0.7 (0.5 to 0.8)	0.07
Aortic annulus, mm*	24 (22 to 25)	23 (22 to 25)	24 (22 to 25)	0.38
Creatinine, µmol/L*	94 (74 to 115)	93 (74 to 11)	96 (76 to 118)	0.21
Estimated glomerular filtration rate, mL/min*	60 (46 to 76)	61 (47 to 77)	60 (46 to 75)	0.36
Haemoglobin, g/L	120±18	127±17	110±13	**<0.001**
Calculated actual PV (mL)	2730±501	2753±509	2693±487	0.17
Calculated actual PV (mL/kg)	38±4	36±3	42±2	**<0.001**
Ideal PV (mL), mean±SD	2842±627	3042±623	2527±488	**<0.001**
PVS, %	−3±10	−9±7	7±6	---
Valve type*				**<0.001**
Sapien XT	199 (36)	121 (35)	78 (36)	
Sapien 3	240 (43)	159 (47)	81 (37)	
Sapien	69 (12)	24 (7)	45 (21)	
Other (eg, Lotus, Portico)	52 (9)	37 (11)	15 (7)	
Valve delivery approach*				**0.002**
Femoral—percutaneous	325 (58)	216 (63)	109 (50)	
Femoral—surgical cut down	95 (17)	56 (16)	39 (18)	
Transapical	140 (25)	69 (20)	71 (32)	
Other	3 (1)	3 (1)	0	
Valve size, mm*	26 (23 to 26)	26 (23 to 26)	26 (23 to 26)	0.71
Volume of contrast, mL*	100 (60 to 120)	100 (65 to 120)	100 (60 to 130)	0.71
Vascular closure technique*				**0.007**
Percutaneous	324 (58)	213 (62)	111 (51)	
Surgical	237 (42)	129 (38)	108 (49)	
Valve-in-valve procedure*	26 (5)	17 (5)	9 (4)	0.64
Blood transfusion*	108 (19)	43 (13)	65 (30)	**<0.001**
Anaesthesia				0.29
General anaesthesia	489 (87)	295 (86)	194 (89)	
Conscious sedation	75 (13)	50 (14)	25 (11)	

Data are n (%), mean±SD, or median (IQR).

P-values <0.05 are highlighted in bold

*Missing values: aortic valve area, 16; aortic valve pathology, 4; blood transfusion, 3; creatinine, 1; coronary artery disease, 3; estimated glomerular filtration rate, 1; EuroSCORE, 23; haemoglobin, 23; hypertension, 2; left ventricular ejection fraction, 6; mean aortic gradient, 24; NYHA class, 1; peak aortic valve gradient, 6; previous cardiac surgery, 1; previous cerebrovascular accident, 2; previous myocardial infarction, 2; previous percutaneous coronary intervention, 4; pulmonary disease, 2; renal failure, 2; severe aortic calcification, 41; vascular closure technique, 3; valve delivery approach, 1; valve-in-valve procedure, 3; valve size, 1; valve type, 4; volume of contrast, 42.

†Creatine >200 umol/L or on dialysis.

EuroSCORE, European System for Cardiac Operative Risk Evaluation; NYHA, New York Heart Association; PV, plasma volume; PVS, plasma volume status.

Relative PV expansion, as defined by a PVS >0%, was evident in 39% of all patients, and in 35% and 42% of all female and male subjects, respectively. Compared with all patients with a PVS ≤0%, those with a PVS >0% were more likely to have a lower body mass index, a logistic EuroSCORE above the median (>14), renal failure, peripheral vascular disease, severe aortic valve calcification, higher mean and peak aortic valve gradients and a lower haemoglobin. On multivariable logistic regression, only a EuroSCORE >14 (OR 1.83, 95% CI 1.27 to 2.62, p<0.001) independently predicted a PVS >0%.

### PVS and mortality after TAVI

**Figure 2:**
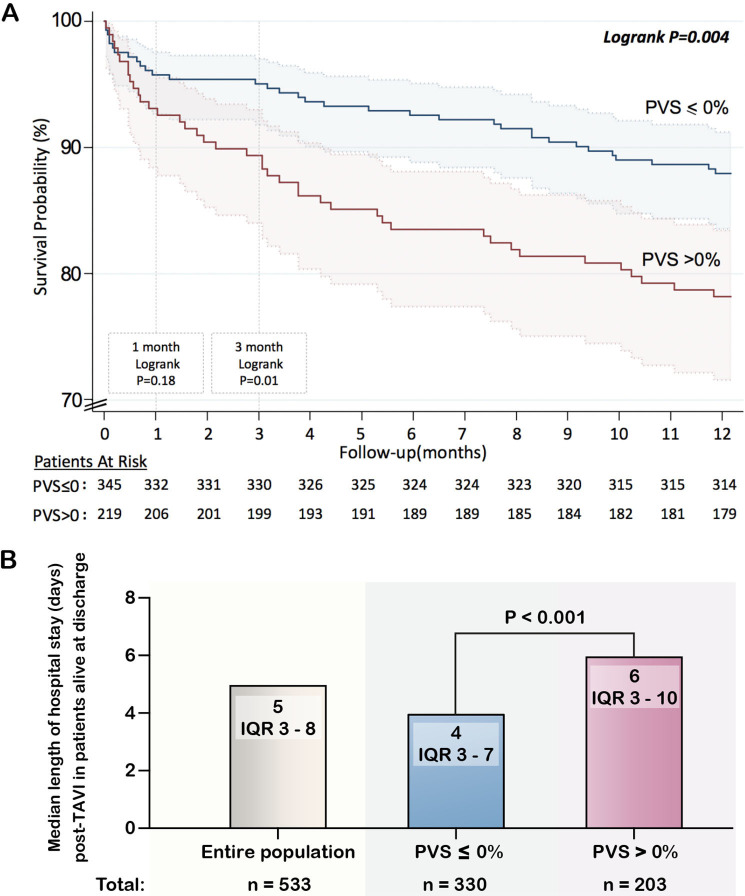
Kaplan Meier survival curve for calculated plasma volume status (PVS) stratified by PVS ≤0% and PVS >0% (A), and duration of hospitalisation after TAVI in patients alive at discharge stratified by PVS ≤0% and PVS >0% (B). The solid line in (A) denotes survival probability and the shaded area in (A) represents the 95% CI. The p value in (B) is calculated from the Mann-Whitney U test. TAVI, transcatheter aortic valve implantation.

After a median hospital stay of 8 (IQR: 3–8) days, 22 (3.9%) patients died in hospital, and 26 (4.6%), 35 (6.2%) and 70 (12.4%) died at 1, 3 and 12 months post-TAVI, respectively. The relation between PVS and the log relative hazard for mortality was non-linear and generally ‘J-shaped’ (figure 1B) at all timepoints. Consequently, PVS was dichotomised using a >0% cut-off as it gave the highest Youden index for discriminating death at all timepoints. A PVS >0% was associated with an increased risk for death at 3 and 12 months after TAVI, but not with inpatient death or mortality at 1 month (table 2) (figure 2A). After adjustment, a PVS >0% was linked to a two-fold heightened risk for mortality at 3 and 12 months, respectively. In a landmark analysis excluding patients who had died by 30 days, a PVS >0% related to 12-month mortality.

**Table 2:** Association of PVS>0% and continuous PVS to deaths and lengths of hospital and intensive care unit stay

	Outcome	Crude HR (95% CI)	P value	Adjusted HR *	P value
				(95% CI)	
**PVS >0%**	In-hospital mortality	1.46 (0.58 to 3.68)	0.43	1.15 (0.44 to 2.96)	0.78
	1-month mortality	2.04 (0.89 to 4.65)	0.09	1.77 (0.77 to 4.07)	0.18
	3-month mortality	2.61 (1.28 to 5.35)	**0.008**	2.29 (1.11 to 4.74)	**0.03**
	12-month mortality	2.20 (1.35 to 3.58)	**0.001**	2.00 (1.23 to 3.26)	**0.006**
	12-month mortality (landmark)	2.35 (1.29 to 4.28)	**0.005**	2.18 (1.19 to 3.99)	**0.01**
	**Outcome**	**Crude regression coefficient (95% CI**)	**P value**	Adjusted * regression coefficient (95% CI)	**P value**
					
**PVS**	Days in the intensive care unit	0.02 (0.00 to 0.04)	**0.01**	0.02 (0.00 to 0.04)	**0.04**
	Days in hospital	0.14 (0.08 to 0.21)	**<0.0001**	0.12 (0.06 to 0.17)	**<0.0001**
					
**PVS >0%**	Days in the intensive care unit	0.48 (0.10 to 0.85)	**0.01**	0.41 (0.04 to 0.78)	**0.03**
	Days in hospital	2.56 (1.03 to 4.10)	**0.001**	1.95 (0.48 to 3.41)	**0.009**

*Adjusted for EuroSCORE >14 and NYHA class ≥3.

EuroSCORE, European System for Cardiac Operative Risk Evaluation; NYHA, New York Heart Association; PVS, plasma volume status.

Addition of PVS >0% to a baseline model incorporating EuroSCORE >14 and NYHA class ≥III incremented model performance as it enabled 11% (95% CI −13% to 35%) of patients dying at 12 months to be correctly reclassified as higher risk, and 28% (95% CI 18% to 37%) of patients surviving to 12 months to be reclassified as lower risk. The overall net reclassification improvement, reflecting the increment in prediction accuracy, was 0.40 (95% CI 0.12 to 0.66). The integrated discrimination index, which reflects the change in calculated risk for each patient was 0.011 (95% CI −0.002 to 0.037) among patients who died, and 0.001 (95% CI −0.000 to 0.005) for patients who survived. The discrimination slope was 1.2 (95% CI −0.2% to 4.2%) percentage points higher than the original.

### PVS and post-TAVI hospitalisation and complications

Median ICU stay (in the total population) and total hospital stay (in patients discharged alive) was 1 (IQR: 0–1) and 5 (IQR 3–8) days, respectively. The relation between PVS and the duration of ICU and hospital stay was essentially linear between PVS values of −15% to 15% (figure 1C). Higher levels of PVS were related to longer ICU and hospital stay, and remained so after adjustment for EuroSCORE >14 and NYHA class ≥III (table 2). Accordingly, patients with a PVS>0% stayed in the ICU and hospital longer after TAVI, compared with patients with a PVS≤0% (figure 2B). A PVS>0% was associated with 0.4 and 2 extra days in ICU and in hospital, respectively, after covariate adjustment (table 2). Addition of continuous PVS to the baseline model enhanced model performance as it enabled 16% (95% CI −7% to 27%) of hospital long stayers to be correctly reclassified as long stayers, and 14% (95% CI 5% to 25%) of short stayers to be reclassified as short stayers. The overall net reclassification improvement was 0.29 (95% CI 0.16 to 0.49). The integrated discrimination index for each patient was 0.015 (95% CI 0.005 to 0.033) among long stayers and 0.014 (95% CI 0.004 to 0.031) for short stayers. The discrimination slope was 3.0 (95% CI 0.9% to 4.2%) percentage points higher than the original.

TAVI was complicated by acute kidney injury (stage II or III), cerebrovascular accident, pacemaker implantation and major vascular injury in 3%, 1%, 8% and 3% of patients, respectively. In unadjusted analyses, continuous PVS was not associated with the risk of acute kidney injury (OR 1.04, 95% CI 1.00 to 1.08, p=0.05), cerebrovascular accident (OR 1.03, 95% CI 0.92 to 1.16, p=0.58), pacemaker implantation (OR 1.02, 95% CI 0.99 to 1.05, p=0.27) or major vascular injury (OR 1.00, 95% CI 0.96 to 1.05, p=0.91). Similarly, a PVS >0% was not associated with the risk of acute kidney injury (OR 1.83, 95% CI 0.88 to 3.84, p=0.11), cerebrovascular accident (OR 3.14, 95% CI 0.28 to 34.87, p=0.35), pacemaker implantation (OR 1.10, 95% CI 0.59 to 2.05, p=0.77) or major vascular injury (OR 0.47, 95% CI 0.15 to 1.47, p=0.20).

## Discussion

Patient optimisation facilitates good TAVI outcomes, and congestion is a correctable adverse factor that can be substantial despite clinical euvolaemia. Using only weights and haematocrits, we evaluated the utility of PVS, a quantitative index of relative volume overload, in TAVI patients. We found that: (1) a relative increase in PV (as defined by a PVS >0%) was present in 39% of patients; (2) a higher logistic EuroSCORE was independently correlated with a PVS >0% and (3) a PVS >0% identified patients at a heightened risk for mortality and prolonged ICU and hospital stay.

Calculated PVS values suggest that our TAVI patients were more congested than stable outpatients with systolic heart failure. This is because mean PVS in our cohort was −3% which is higher (ie, more congested) than the −9% reported in 5002 outpatients with chronic heart failure with reduced ejection fraction (HFrEF).^16^ This is clinically plausible and likely reflects the fact that TAVI patients are sicker, commonly have HFrEF or heart failure with preserved ejection fraction (HFpEF) and are frequently on suboptimal doses of heart failure drugs due to blood pressure and/or renal function limitations. Indeed, higher doses of prognostic medications are linked to higher odds of having an optimal PVS in patients with HFrEF.^17^

That a higher logistic EuroSCORE was the only independent correlate of PV expansion likely mirrors the fact that it is derived from many variables that drive congestion. This includes left ventricular systolic dysfunction, diabetes, a critical preprocedural clinical status, pulmonary hypertension and renal dysfunction. Despite this association, however, a PVS >0% provided prognostic information that was independent and complementary to that offered by the EuroSCORE. It is important to note that only 3% of our cohort had renal failure (defined as creatinine >200 μmol/L or requiring dialysis), and it is conceivable that if the number of patients with renal failure were larger, it may have been a multivariable predictor pf PVS >0%.

Calculated PV expansion related to worse outcomes after TAVI, in line with prior data in chronic HFrEF patients^16 17 19^ and other cohorts^18 20 22^ In 1887, patients undergoing coronary artery bypass grafting, a preoperative PVS ≥5.6% was linked to a twofold increase in in-hospital mortality, longer hospital stays and greater postoperative renal and arrhythmic complications.^20^ In 3414 HFpEF patients, each 5% increment in PVS was associated with an ~11% higher risk of death or heart failure hospitalisation.^19^ Moreover, in 1115 patients hospitalised for acute HFrEF, each 1% increment in admission PVS forecasted a 21% increased risk for death.^18^

In a cohort of 652 TAVI patients, PVS ≥4 was associated with all-cause mortality in the longer term and was associated with a 30-day composite of all-cause mortality, stroke, life-threatening bleeding, acute kidney injury, coronary artery obstruction requiring intervention, major vascular complication and valve related dysfunction requiring repeat procedure.^22^ However, PVS ≥4 was not associated with the primary endpoint for that study,^22^ that is, 30-day mortality, and until now the findings have not undergone external validation in an independent cohort. Our study builds on previous data, by independently validating the association between elevated PVS and mortality, and showing that PVS >0% is associated with prolonged ICU stay.

The adverse implications of a PVS >0% in TAVI patients most likely reflects the ominous consequences of PV expansion in a cohort with stiff non-compliant ventricles. This further exaggerates increases in intravascular filling pressures per unit change in ventricular volumes, leading to a greater propensity to pulmonary and systemic oedema, multiorgan hypoperfusion and adverse outcomes after TAVI. Alternatively, it could be argued that the PVS equation does not truly gauge PV but merely reflects the prognostic implications of weight and haematocrit, or other factors in TAVI cohorts that modulate these variables. For example, patients with PVS >0% had lower body mass index, potentially reflecting frailty, which is known to predict long-term mortality after TAVI.^11^ However, calculated PV levels have been shown to correlate well to PV levels measured using gold-standard radioisotope assays,^16 17^ and calculated PVS is known to remain prognostic even after extensive covariate adjustment including for natriuretic peptides.^16–19^

Our results should be interpreted in the context of study limitations. First, this is an observational study, so causality cannot be inferred. Second, the median length of hospitalisation in our cohort is relatively long as it included patients who had TAVI as far back as 2007, whereas, in the current era, duration of hospitalisation is on average shorter due to improvements in patient selection and technical advances. Third, information on natriuretic peptides and clinical signs of congestion was unavailable. Fourth, even though patients with PVS >0% had higher incidence of blood transfusions and transapical approach during TAVI, and we expected to observe higher short-mortality in this group of patients, the relatively small number of deaths in hospital and by 30 days post-TAVI limited the power to detect statistically significant mortality differences at this timepoint. Similarly, few patients had acute kidney injury stage II or III following TAVI, so we are likely underpowered for this endpoint.

Despite limitations, our study has potentially important ramifications. First, it suggests that calculated PVS might be of clinical utility in guiding objective fluid management decisions before and after TAVI. Diuretic and non-diuretic therapy could then be titrated to keep PVS≤0% irrespective of apparent clinical euvolaemia. Because calculated PVS relies only on weight and haematocrit, it provides a potentially simple non-invasive means of gauging congestion that could have wide applicability. Second, our data justify incorporation of PVS into risk-stratification models in patients undergoing TAVI, which could potentially help identify patients for early discharge, or for more intensive monitoring after TAVI. Third, our data suggest that further observational and interventional studies may be warranted to objectively determine the clinical utility of PVS-guided optimisation of TAVI patients. We did not measure PVS after TAVI, hence it was not possible to measure change in PVS after the procedure relative to baseline, which might be relevant for future research, because the relief of aortic stenosis would be expected to reduce PVS.

In conclusion, a PVS >0%, calculated simply from weight and haematocrit, is associated with a higher logistic EuroSCORE, but independently and incrementally predicts an increased risk of mortality and prolonged hospitalisation after TAVI. Prospective evaluation of the utility of calculated PVS-guided optimisation of TAVI patients is warranted.
